# Deep Learning and Geometry Flow Vector Using Estimating Vehicle Cuboid Technology in a Monovision Environment

**DOI:** 10.3390/s23177504

**Published:** 2023-08-29

**Authors:** Byeongjoon Noh, Tengfeng Lin, Sungju Lee, Taikyeong Jeong

**Affiliations:** 1 Department of AI and Big Data, Soonchunhyang University, 22 Soonchunhyang-ro, Asan 31538, Republic of Korea; powernoh@sch.ac.kr; 2 Department of Civil and Environmental Engineering, Korea Advanced Institute of Science and Technology, 291 Daehak-ro, Yuseong-gu, Daejeon 34141, Republic of Korea; tengfenglin@kaist.ac.kr; 3 Department of Software, Sangmyung University, Cheonan 31066, Republic of Korea; 4 School of Artificial Intelligence Convergence, Hallym University, Chuncheon 24252, Republic of Korea

**Keywords:** cuboid detection, object detection, deep learning, road vehicle detection, road geometry

## Abstract

This study introduces a novel model for accurately estimating the cuboid of a road vehicle using a monovision sensor and road geometry information. By leveraging object detection models and core vectors, the proposed model overcomes the limitations of multi-sensor setups and provides a cost-effective solution. The model demonstrates promising results in accurately estimating cuboids by utilizing the magnitudes of core vectors and considering the average ratio of distances. This research contributes to the field of intelligent transportation by offering a practical and efficient approach to 3D bounding box estimation using monovision sensors. We validated feasibility and applicability are through real-world road images captured by CCTV cameras.

## 1. Introduction

The rapid progress in information and communication technology (ICT) has prompted cities worldwide to embrace the concept of smart cities, leveraging intelligent platforms to enhance various aspects of urban life [1,2,3,4,5]. In particular, the field of intelligent transportation has witnessed significant transformations, aiming to automatically gather crucial information about vehicle positions, speeds, and types using vision sensors [6,7]. These data play a pivotal role in enabling intelligent signal control [8], analyzing traffic risk behavior [1,9], predicting traffic states [10], and more [3,11,12]. Consequently, there is a growing emphasis on the utilization of vision sensors to detect, track, and identify road users.

Meanwhile, object detection stands as a fundamental challenge in the realm of computer vision [13]. Over the past few years, extensive research has been conducted in 2D object detection using deep learning models such as convolutional neural networks (CNNs), which are widely used deep learning models, and their applications [14,15,16,17]. Nowadays, the You Only Look Once (YOLO) series models are frequently used to detect objects in images [13,18]. For example, in 2016, Redmon et al. pioneered a unique convolutional network that simultaneously predicts multiple bounding boxes and their associated class probabilities, enhancing performance speed [19]. Fast forward to 2018, the YOLOv3 model was developed by building on YOLO’s foundation, addressing the challenge of detecting small-scale objects and achieving a balance between efficiency and accuracy [18]. Bochkovskiy et al., in an effort to optimize the performance of high frames-per-second (FPS) videos, engineered the YOLOv4 model, introducing improvements to the backbone, activation function, and loss function [20]. More recently, YOLOv7 has been launched, dramatically improving detection accuracy without escalating the inference cost for real-time object detection. In parallel, Tian et al. proposed a revolutionary framework, FCOS, that eliminates the need for proposals and anchors, significantly cutting down the number of design parameters [21,22].

Recent focus has shifted towards a more fundamental challenge in the field: the accurate estimation of 3D bounding boxes for vehicles within the surrounding environment. Ensuring the precise perception of spatial dimensions is crucial for a multitude of applications, including object detection, tracking, and collision avoidance. Traditionally, the estimation of 3D bounding boxes has relied on multi-sensor setups, such as the integration of multiple RGB cameras or the fusion of camera and Light Detection and Ranging (LiDAR) sensors [23]. Significant literature has explored the utilization of multiple RGB cameras [24], RGB-D cameras (such as Microsoft Kinect) [25], and sensor fusion techniques [26] for accurate 3D bounding box recognition. For example, Li et al. made significant contributions with their introduction of a 3D object detection model termed Stereo R-CNN, a model that is based on stereovision techniques [24]. Striving for superior performance, Ku et al. advanced a distinctive model named AVOD. This model taps into the potential of LiDAR point clouds and RGB images to undertake a variety of tasks, including 3D localization, orientation estimation, and category classification [26]. Building on the versatility of RGB-D images, Deng et al. utilized RGB-Depth Images for object detection. Their approach aims to identify object locations within the 3D environment and gauge their physical dimensions and pose, proving effective even in situations where objects overlap [25].

However, these configurations come with significant costs, calibration complexities, maintenance requirements, and the absence of large-scale annotated datasets tailored for multiple sensing-based approaches, similar to the impact of ImageNet in 2D object detection [27]. Even though the expenditure associated with a stereo camera is not as hefty as that of LiDAR, it still presents challenges, such as the complexity of calibration and the need for extensive work on stereo matching to minimize disparity errors [28]. The maintenance costs for LiDAR and stereo cameras are considerably higher compared to Closed-Circuit Television (CCTV) cameras [29]. As a result, there has been a rising demand for research on 3D object detection using a single-vision sensor. Researchers have explored pioneering approaches to address 3D object detection using monocular cameras. For instance, Li and his team introduced Mono3D, a system that leverages a mono-camera to employ segmentation, context, and location cues for precise 3D object detection [29]. Similarly, Chabot and colleagues proposed a two-step approach that predicts 2D boxes, vehicle geometry, and visibility attributes first, followed by recovering 3D orientations and locations [30]. Mousavian and his team advanced a simplified network architecture for 3D object prediction, utilizing projective geometry constraints for 3D object bounding box detection and employing deep CNN for object orientation and size regression [31].

Despite the promising performance of current research based on monocular cameras, a significant constraint remains: the need for a high-quality labeled dataset specifically for model training. The model’s performance relies heavily on the quality of the 3D labeled dataset. Therefore, it is crucial to explore more efficient and low computational complexity methods for handling 3D object detection. In summary, while multi-sensor setups offer advantages in certain scenarios, they also come with various challenges and costs. The emerging research on 3D object detection using monocular cameras has yielded promising results, but the need for high-quality labeled datasets remains a critical aspect to be addressed for further improvements. Efficient and low computational complexity techniques are essential to enhance the performance of 3D object detection using single vision sensors.

In this study, we propose a novel model named “*Vehiclectron*” for accurately estimating the 3D bounding boxes (cuboids) of vehicles on roads. Our model leverages a monovision sensor, specifically a CCTV camera, along with road geometry information. To begin, the proposed model detects vehicles in video footage using object detection models. Subsequently, three core vectors are extracted: the flow vector, orthogonal vector, and perpendicular vector. The flow vector represents the front direction of the vehicle, which is determined by considering the directions of traffic flow and road geometry. The orthogonal vector is perpendicular to the flow vector and serves as the basis for the cuboid orthogonal to the vehicle. Lastly, the perpendicular vector points vertically downwards within the vehicle in the virtual *z*-axis of the 2D image.

By utilizing these three core vectors, the directions of the eight edges in the vehicle’s cuboid can be determined. To estimate the magnitudes of these edges, we employ the average ratio of distances between the center points of the 2D bounding box for each object and the corresponding vertex in the 3D bounding box (ground truth). Consequently, the 3D bounding box of the vehicles in the 2D image can be accurately estimated using only the monovision sensor.

Our proposed *Vehiclectron* model presents a valuable contribution as it overcomes the limitations of traditional approaches that rely on multi-sensor setups and demonstrates the potential for accurate 3D bounding box (cuboid) estimation using a monovision sensor and road geometry information. By harnessing the capabilities of a monovision sensor such as CCTV, this model offers a cost-effective and easily deployable solution for estimating the 3D bounding boxes of vehicles. We confirm the feasibility and applicability of the proposed model by applying it to vehicles in actual road images from CCTVs of multiple roads.

## 2. Materials and Methods

### 2.1. Data Sources

For our experiment, we obtained road vehicle images from the AI-Hub platform [32], which serves as a data repository for sharing datasets with model developers who train their artificial intelligence models. This platform allows users to access publicly available data, including text (such as corpus data and machine reading comprehension data) as well as vision data (such as video, image, eye gaze, and traffic data). In this particular study, we utilized approximately 117,926 images containing around 724,196 road vehicles in Bucheon City, Republic of Korea. Among these vehicles, there were approximately 62,332 trucks, 40,104 buses, 607,030 general cars, 17,730 bikes, and 1143 unknown objects. An unknown object refers to an object that does not fall within the predefined categories of vehicle types. This could be because the object belongs to a special category such as excavator or other unique vehicles. It could also be due to the object being relatively small in size or appearing blurry or overlapped in the image or video frames, often a result of being located at a significant distance from the camera.

**Figure 1:**
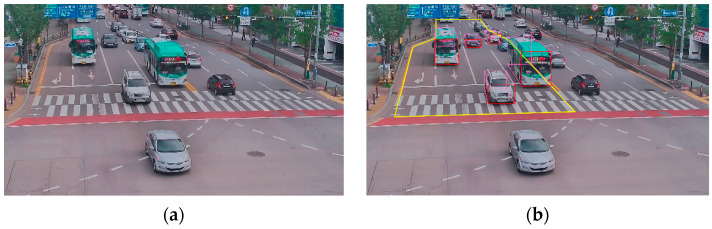
Examples of used (**a**) original image; and (**b**) 3D bounding box-annotated image.

**Figure 2:**
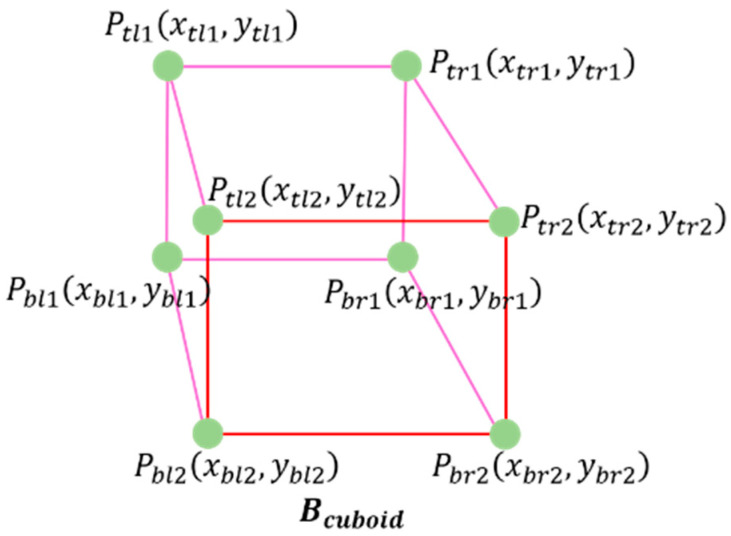
Notation of x–y coordinates in $V_{cuboid}$.

The utilized dataset includes annotations for cuboids, consisting of eight pairs of x–y coordinates, as depicted in Figure 1. Figure 1a,b represent the original image and the annotated image, respectively. In Figure 1b, the yellow box denotes the region of interest (RoI) used to capture the traffic flow, which is essential for calculating the flow vector. The annotated data are provided as XML, and consist of various image metadata, such as the image size, file name, and location ID. Additionally, there are eight pairs of x–y coordinates for cuboids, $V_{cuboid}$, in the local image plane, namely $P_{tl1}\left(x_{tl1},y_{tl1}\right)$, $P_{bl1}\left(x_{bl1},y_{bl1}\right)$, $P_{tr1}\left(x_{tr1},y_{tr1}\right)$, $P_{br1}\left(x_{br1},y_{br1}\right)$, $P_{tl2}\left(x_{tl2},y_{tl2}\right)$, $P_{bl2}\left(x_{bl2},y_{bl2}\right)$, $P_{tr2}\left(x_{tr2},y_{tr2}\right)$, $P_{br2}\left(x_{br2},y_{br2}\right)$, as illustrated in Figure 2. These coordinates correspond to the top-left and bottom-right positions of the cuboid, and the class of the vehicle type is also represented. Specifically, ‘xtl1’ and ‘ytl1’ refer to the top-left coordinates on the bottom side of the cuboid, while ‘xbr2’ and ‘ybr2’ represent the bottom-right coordinates on the top side of the cuboid.

### 2.2. Proposed Methods

The proposed model consists of three primary components: (1) object detection, (2) core vector extraction, and (3) cuboid estimation. In the object detection stage, deep learning-based algorithms such as the YOLO-series or faster/mask R-CNN models are employed to detect various objects, particularly road vehicles. Subsequently, utilizing the center points extracted from the 2D bounding boxes obtained through object detection, three core vectors are derived to estimate the cuboid of the road vehicle. Finally, the cuboid is estimated based on the outcomes of the object detection model and core vector extraction. This proposed model comprises these interconnected stages to achieve the accurate estimation of the cuboids of road vehicles.

#### 2.2.1. Object Detection Using Deep Learning Model

Our primary objective at this stage is to identify road vehicles in the 2D space of the image, particularly the center points within the 2D bounding boxes, which are crucial for estimating the corresponding 3D bounding boxes. To accomplish this, we utilize YOLOv7 [13], the latest iteration in the YOLO series, which incorporates significant improvements to the detector heads and backbone network. YOLOv7 benefits from the trainable bag-of-freebies techniques, which enhances real-time object detection accuracy without increasing the computational cost. Furthermore, this model offers the advantages of reducing parameter and computation requirements. These enhancements make this highly effective and accurate, making it a valuable tool for object detection in our proposed system. Additionally, its streamlined and lightweight architecture enables seamless integration with edge devices, such as CCTV systems, to enable real-time detection capabilities.

Hence, we employ the YOLOv7 network to detect road vehicles in our proposed model, and the outputs include the specific class of the detected object, and bounding box information, ${\hat{B}}_{box}$, such as the x and y coordinates of the object’s center point (measured in pixels). The information of the estimated bounding box is used to obtain the core vectors and size of cuboids in our proposed system.

#### 2.2.2. Core Vector Extraction

In this section, we describe the process to extract three core vectors (flow vector, orthogonal vector, and perpendicular vector) to estimate the cuboid of a road vehicle. Note that each core vector is extracted at the site level. In the cuboid estimation step, we can obtain the complete cuboid from the 2D bounding box in the object detection model and the three core vectors.

**Figure 3:**
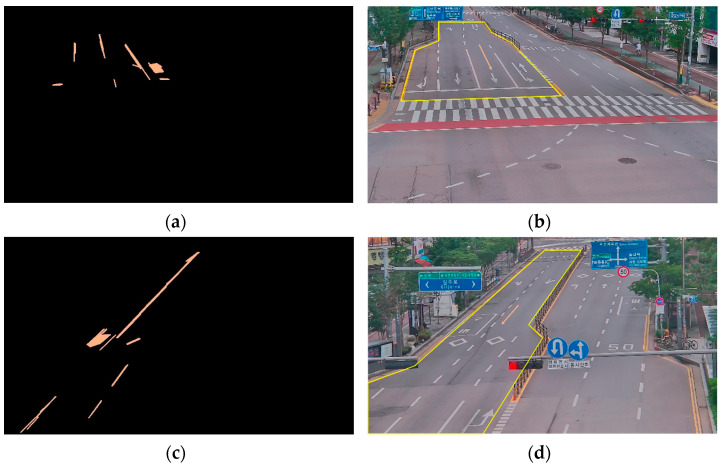

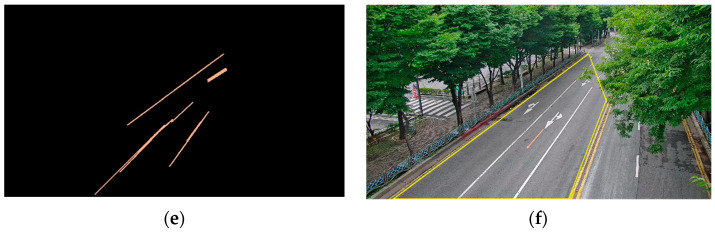
Examples of the extracted lanes (**a**,**c**,**e**) and flow vectors (**b**,**d**,**f**), respectively.

**Flow vector extraction** The flow vector is decided by taking into account the directions of traffic flow and road geometry. The underlying assumption is that vehicles generally adhere to the lanes on the road. Therefore, within the designated region of interest (RoI), we detect the lanes and extract the primary vector that represents the front direction of the vehicle. This is achieved through the utilization of the Hough transform [33,34,35], a widely used feature extraction technique in the fields of computer vision and pattern recognition. The Hough transform algorithm operates by converting the Cartesian coordinates (x, y) of image points into a parameter space known as the Hough space, denoted by (ρ, θ) [36,37]. Within the Hough space, each image point corresponds to a curve or sinusoid, which represents a potential shape or pattern. Prior to applying the Hough transform, an initial step of edge detection is typically performed to identify pertinent features such as lines or curves. In our experiment, our focus is specifically on detecting the road lanes, allowing us to isolate the desired features for detection purposes. The Hough transform functions as a voting process, where each point belonging to potential patterns contributes votes to all the possible patterns passing through that point. These votes are accumulated in an accumulator array, referred to as bins, and the pattern with the highest vote count is recognized as the desired pattern. As a result of the Hough transform, we are able to obtain multiple lanes within the RoI, and by calculating the average of the obtained lane vectors, we can determine the flow vector at that specific location. For visual reference, Figure 3a,c,e show the directional edges of lanes using the edge detection and Hough transform algorithms. A set of the directional edges in spot i is notated as $D_{f}^{i}=\left[{\vec{d}}_{f,1}^{i},\;{\vec{d}}_{f,2}^{i},\ldots ,{\vec{d}}_{f,n}^{i}\right]$, where n is the number of directional edges in the given spot. The flow vector $\vec{f_{i}}$ is only directional information, and not the magnitude of the vector, which is the mean of $D_{f}^{i}$. Figure 3b,d,f show the corresponding directions of the flow vectors $\vec{f_{i}}$ in each spot.

**Figure 4:**
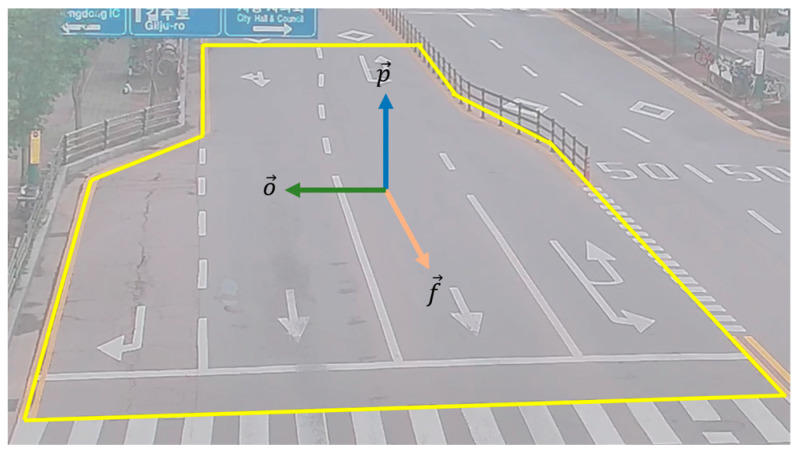
Representation of flow vector ($\vec{f}$), orthogonal vector ($\vec{o}$), and perpendicular vector ($\vec{p}$).

**Estimation of orthogonal and perpendicular vectors** Following the extraction of the flow vector at each site, we proceed to estimate the orthogonal and perpendicular vectors. The orthogonal vector is perpendicular to the flow vector and acts as the foundation for the cuboid orthogonal to the vehicle. In contrast, the perpendicular vector extends vertically downwards within the vehicle along the virtual *z*-axis of the 2D image. These vectors play a crucial role in determining the spatial dimensions and orientation of the vehicle cuboid. Figure 4 illustrates the examples of the extracted vectors for constructing the cuboid; flow vector ($\vec{f}$), orthogonal vector ($\vec{o}$), and perpendicular vector ($\vec{p}$).

In general, vector consists of the direction and magnitude; $\vec{o}=\left\{\theta_{o},\;\left|\vec{o}\right|\right\}$ and $\vec{p}=\left\{\theta_{p},\;\left|\vec{p}\right|\right\}$, so we first calculate the directions of $\vec{o}$ and $\vec{p}$. For this, we compute the average angles, representing the directions, between the acquired $\vec{f_{i}}$ and the edges within the ground truth cuboid $B_{cuboid}$ in spot i. $\theta_{o}^{i}$, is calculated by $\vec{f_{i}}$ and a set of the horizontal edge vectors in cuboid j in spot i, denoted as $H_{i,\;j}=\left[\vec{P_{tl1}^{i,j}P_{tr1}^{i,j}},\;\vec{P_{tl2}^{i,j}P_{tr2}^{i,j}},\;\vec{P_{bl1}^{i,j}P_{br1}^{i,j}},\;\vec{P_{bl2}^{i,j}P_{br2}^{i,j}}\right]$, as follows:

$$\theta_{o}^{i}=\frac{\sum_{j=1}^{T}g_{H_{i,j}}\left(\vec{f_{i}}\;\right)}{T}$$

$$g_{H_{i,j}}\left(\vec{f_{i}}\;\right)=\frac{\sum_{\vec{P^{i,j}}\in H_{i,\;j}}\arccos\left(\left(\vec{P^{i,j}}\cdot \vec{f_{i}}\right)/\left(\left\|\vec{P^{i,j}}\|\times \|\vec{f_{i}}\right\|\right)\right)}{\left|H_{i,\;j}\right|}$$

where T is the number of total vehicles in spot i. Similarly, $\theta_{p}^{i}$ is calculated with $\vec{f_{i}}$ and a set of the vertical edge vectors in cuboid j in spot i, denoted as $V_{i,\;j}=\left[\vec{P_{bl1}^{i,j}P_{tl1}^{i,j}},\;\vec{P_{br1}^{i,j}P_{tr1}^{i,j}},\;\vec{P_{bl2}^{i,j}P_{tl2}^{i,j}},\;\vec{P_{br2}^{i,j}P_{tr2}^{i,j}}\right]$, as follows:

$$\theta_{p}^{i}=\frac{\sum_{j=1}^{T}g_{V_{i,j}}\left(\vec{f_{i}}\;\right)}{T}$$

$$g_{V_{i,j}}\left(\vec{f_{i}}\;\right)=\frac{\sum_{\vec{P^{i,j}}\in V_{i,\;j}}\arccos\left(\left(\vec{P^{i,j}}\cdot \vec{f_{i}}\right)/\left(\left\|\vec{P^{i,j}}\|\times \|\vec{f_{i}}\right\|\right)\right)}{\left|V_{i,\;j}\right|}$$

**Figure 5:**
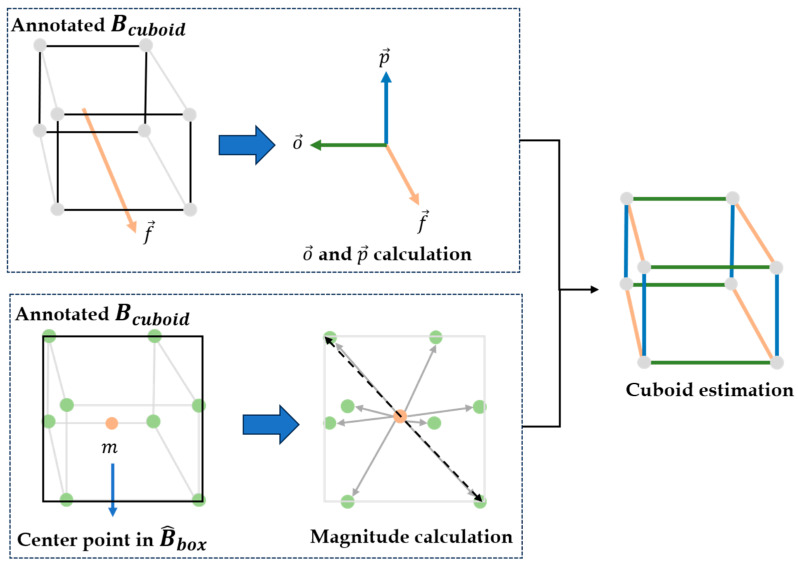
Overall process for estimating cuboid using flow vectors, ${\hat{B}}_{box}$ and $B_{cuboid}$.

Next, the magnitude of ${\vec{o}}_{i}$ and ${\vec{p}}_{i}$ are obtained by each ratio of distances between center point $\hat{m}$ in ${\hat{B}}_{box}$ and eight vertices in $B_{cuboid}$ and a length of diagonal line in ${\hat{B}}_{box}$, which assumes that the size of the bounding box is proportional to the size of the cuboid. Thus, by utilizing them, we can estimate the size of the cuboid when the objects are detected in the object detection model. Combining these distance ratios with the core vectors allows us to estimate the complete form of the cuboid. In detail, we compute the ratios of distances from the center point to each vertex considering the specific class of road vehicle, denoted as c. It is important to note that different vehicle types have varying sizes; hence, there is differentiation in computations based on the vehicle class. The ratios of distances by cuboid’s vertex and class in spot i are calculated as follows:

$$r_{c}^{i}=\frac{d_{c}^{i}}{l_{c}^{i}};\;d_{c}^{i}=\left\{\sum_{j=1}^{T_{c}}dist\left({\hat{m}}_{i,\;c},\;P_{*}^{i,\;c}\right)|\;P_{*}\in B_{cuboid}\right\}$$

where $l_{c}^{i}$ is the average length of the diagonal line in the bounding box. As a result, we can obtain $\left|\vec{o}\right|$ and $\left|\vec{p}\right|$ by using $d_{c}^{i}$. Figure 5 depicts the overall process for estimating the vehicle’s cuboid with flow vectors, ${\hat{B}}_{box}$ and $B_{cuboid}$.

## 3. Experiments and Results

### 3.1. Experimental Design

This section presents the experimental design used to evaluate the proposed model for estimating the cuboid of road vehicles using an object detection algorithm and core vector calculation. The study consists of two main experiments with the following objectives:

(1) To detect road vehicles in video footage and determine their 2D bounding boxes using various object detection models. The aim is to select the most optimal object detection model for this task.
(2) To evaluate the accuracy of the estimated cuboid using core vectors by 3D IoU (Intersection on Union) as the evaluation metric.

The experimental design involves conducting these three experiments to achieve the specified objectives. The results obtained from each experiment will contribute to the overall evaluation of the proposed model.

Meanwhile, a typical IoU is a metric used to assess the accuracy of object detection, instance segmentation, and semantic segmentation algorithms in computer vision tasks in 2D image [38]. It quantifies the spatial similarity between a predicted region and a ground truth region, providing a measure of localization and segmentation accuracy. IoU is defined as the ratio of the area of intersection between the predicted and ground truth regions to the area of their union. The resulting value ranges from 0 to 1, where 0 indicates no overlap between the regions, and 1 signifies a perfect match. The IoU serves as an evaluation metric, with higher values indicating better algorithm performance in terms of localization and segmentation accuracy. Lower IoU values, however, suggest poorer performance. By comparing the predicted and ground truth regions, IoU provides a quantitative measure of how well an algorithm is performing, enabling researchers and practitioners to assess and improve the quality of their models. The formula is as follows:

$$IoU=\frac{\left(Area\;of\;Intersection\right)}{\left(Area\;of\;Union\right)}$$

In this study, we have employed the 3D IoU as an evaluation metric for assessing the accuracy of the estimated cuboid. The 3D IoU extends the concept of IoU from 2D images to a 3D perspective. It takes into account the IoUs of all sides of the cuboid. Let I be a set of IoUs in all sides of the cuboid, where the cardinality of the set is 6 for a cuboid. The 3D IoU is calculated as the average of the IoUs in all sides of the cuboid, using the following formula:

$$3D\;IoU=\frac{1}{\left|I\right|}\underset{IoU^{*}\in I}{\sum }IoU^{*}$$

By averaging the IoUs of all sides, the 3D IoU provides a comprehensive measure of the spatial similarity between the estimated cuboid and the ground truth, enabling an accurate assessment of the localization and segmentation performance in a 3D context.

### 3.2. Results

This section focuses on validating the performance of the object detection model used, YOLOv7, and the results of cuboid estimation. First, we utilized the COCO-pretrained model and trained it on 70% of the available data, reserving the remaining 30% for testing purposes in our experiment. The dimensions of the input image are 1920 × 1080 pixels, which are subsequently resized to 640 × 640 pixels to serve as the input shape for the YOLOv7 model. The evaluation metrics employed are accuracy and mIoU between the ground truth and the estimated bounding boxes by YOLOv7. Accuracy is determined by comparing the number of vehicles in the ground truth with the estimated results. Additionally, mIoU are computed based on the information provided by the ground truth and the estimated bounding boxes for each class.

The results of this experiment are presented in Table 1, indicating an average detection accuracy of approximately 0.98 and mIoU of about 0.871. These values demonstrate the effectiveness of the YOLOv7 object detection model in accurately identifying road vehicles in the video footage.

**Figure 6:**
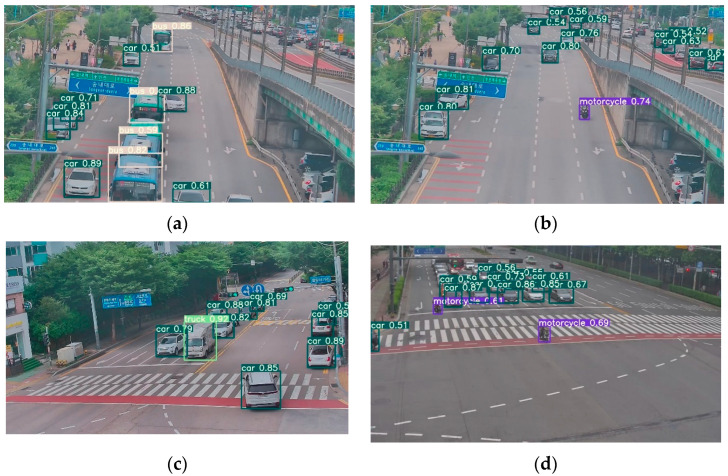
(**a**–**d**) Results of object detection model in our experiment using YOLOv7.

Figure 6a–d show the sample images illustrating the results of the object detection model in the experiment. This model successfully detects the majority of road vehicles, such as general vehicles (annotated as ‘car’), bus, truck, and bike (annotated as ‘motorcycle’). Upon examining the images, it becomes evident that the object detection model effectively identifies road vehicles. Nevertheless, in some instances, certain objects might be partially or completely obscured by other objects present in the scene. This occlusion can result in the model failing to detect the entire extent of the obscured vehicle.

**Figure 7:**
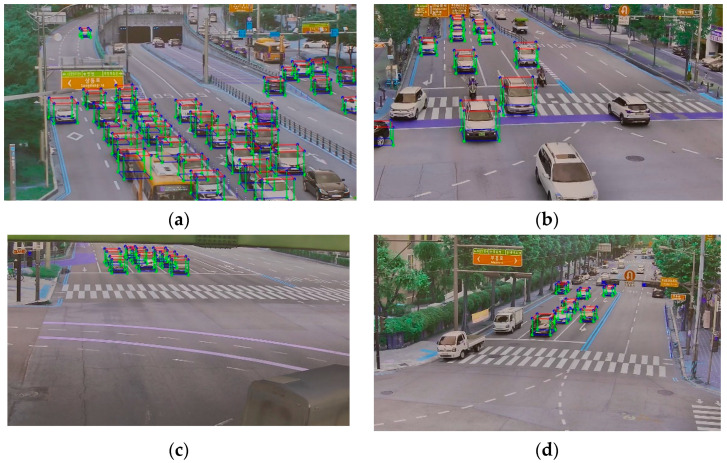
(**a**–**d**) Results the estimated cuboids in some different sites.

Next, we proceeded with the validation of the results obtained from the cuboid estimation. To evaluate the accuracy of the estimated cuboids, we employed the 3D IoU as the evaluation metric, with an average 3D IoU value of 0.89. Figure 7 illustrates some examples of the estimated cuboids in various locations. The visual inspection suggests a close match between the estimated cuboids and the actual objects.

These findings indicate that the estimated cuboids align well with the ground truth, as indicated by the high 3D IoU values and visual agreement was observed.

### 3.3. Discussion

The proposed model aims to estimate the cuboid of road vehicles in a 3D context using a vision-based object detection model and core vectors. The YOLOv7 model, a general object detection model, is utilized to detect road vehicles in 2D images based on their classes. Additionally, core vectors are derived from the road geometry, assuming that traffic flow is parallel to the lane direction. The flow vector is obtained through the edge detection and Hough transform algorithms, while orthogonal and perpendicular vectors are extracted based on the vehicle’s location and type. These core vectors contain directional information only. The size of the cuboid, specifically the lengths of its edges, is calculated using the magnitudes of these vectors, derived from the ratios of the distances between the diagonal line in bounding box and the center point obtained from object detection and each vertex in the annotated cuboid. By utilizing these core vectors, the complete cuboid of road vehicles can be drawn.

Through a series of experiments, the effectiveness of the system is evaluated both quantitatively and qualitatively. The first experiment focuses on validating the object detection model used, demonstrating its effective detection of road vehicles in images. The second experiment involves estimating the actual cuboid using the detected bounding box information and flow vector. The results are validated using the 3D IoU as the evaluation metric.

It is worth noting that the proposed model has certain limitations, such as its dependence on the geometric information of the site and the object detection model. For example, in Figure 7b,d, some bikes and trucks were not detected. However, to the best of our knowledge, this model represents a pioneering attempt in utilizing a monovision sensor alone to estimate cuboids in a 3D context. Furthermore, the proposed system can be applied to stationary video sensors, such as CCTVs, for traffic flow recognition and management, where precise information regarding vehicle coordinates and sizes is required.

## 4. Conclusions

In this study, we proposed a model named “*Vehiclectron*” for estimating the cuboid of road vehicles in a 3D context by combining a vision-based object detection model and core vectors. The utilization of the YOLOv7 object detection model proved effective in accurately detecting road vehicles in 2D images. The core vectors, derived from road geometry and flow information, played a crucial role in estimating the cuboid by providing essential directional information. Our experimental results, validated using the 3D Intersection over Union (IoU) metric, demonstrated the effectiveness of the proposed model with an average 3D IoU value of 0.89. Furthermore, we conducted practical implementation and application of the proposed model using various road vehicles in different real-world sites in Bucheon City, Republic of Korea. This validation confirmed the feasibility and applicability of our model.

However, since the proposed model estimates the cuboids of the road vehicles by using the 2D bounding box and core vectors from road geometry, there are some limitations that the 3D cuboid estimation has a dependency on the results from the object detection model (YOLOv7 in our case) in terms of accuracy and inference time. The objects may be partially or entirely obscured by other nearby objects, leading to reduced detection accuracy. Additionally, the detection of small-sized vehicles located far from the CCTV camera remains a challenge. The reduced resolution and distance from the camera can hinder the model’s ability to accurately recognize and classify such vehicles. However, it is essential to consider that the target vehicles in our model are those approaching the camera, not those moving away in other lanes. Consequently, these vehicles may eventually become more visible and detectable as they come closer to the camera, and their size in the image increases.

From the perspective of inference time, our model’s performance is commendable in GPU environments, with processing time, including inference time and core vector computing time, typically below 0.1 s per frame. While this is a respectable frame rate, it falls short of real-time processing capabilities. To address the limitation in achieving true real-time performance, further optimizations and hardware considerations may be necessary. However, it is important to note that our proposed model utilizes a single vision sensor, eliminating the need for data processing from multiple sensors and avoids the time-consuming calibration process required by multi-camera systems for cuboid estimation. While multi-camera systems may achieve higher accuracy in certain cases, they often come with the trade-off of increased computational complexity and inference time. In contrast, our proposed model strikes a balance between accuracy and inference speed, making it well-suited for dynamic and time-sensitive applications.

Overall, our proposed model represents a novel approach to estimating cuboids using a monovision sensor in 2D images combined with road geometry and core vectors. We believe that, in the future, this model holds potential for providing precise information in the field of intelligent traffic recognition and control. Ongoing research and development efforts are focused on advancing this model to further enhance its capabilities and broaden its application scope.

## Figures and Tables

**Table 1:** The performance of the object detection model. # means “the number of”.

	Class	# of Objects in Test	Accuracy	mIoU
**Ground truth**	General vehicle	182,109	-	-
	Bus	12,031	-	-
	Trucks	18,699	-	-
	Bikes	5319	-	-
**Object** **detection model (YOLOv7)**	General vehicle	179,377	0.985	0.871
	Bus	11,429	0.950	0.910
	Trucks	18,025	0.964	0.870
	Bikes	4584	0.862	0.774
	**Average**		**0.978**	**0.871**

## Data Availability

Data sharing not applicable.
